# The genome sequence of the northern goshawk, *Accipiter gentilis *(Linnaeus, 1758)

**DOI:** 10.12688/wellcomeopenres.17821.1

**Published:** 2022-04-04

**Authors:** Katherine August, Martin Davison, Chiara Bortoluzzi, Wellcome Sanger Institute Tree of Life programme, Wellcome Sanger Institute Scientific Operations: DNA Pipelines collective, Tree of Life Core Informatics collective, Darwin Tree of Life Consortium

**Affiliations:** 1 University of Aberdeen, Aberdeen, UK; 2 Northumberland and Tyneside Bird Club, Northumberland, UK; 3 Tree of Life, Wellcome Sanger Institute, Cambridge, UK

**Keywords:** Accipiter gentilis, northern goshawk, genome sequence, chromosomal, Aves

## Abstract

We present a genome assembly from an individual female *Accipiter gentilis *(the northern goshawk; Chordata; Aves; Accipitriformes; Accipitridae). The genome sequence is 1,398 megabases in span. The majority of the assembly (99.98%) is scaffolded into 40 chromosomal pseudomolecules, with the W and Z chromosomes assembled. The complete mitochondrial genome was also assembled and is 16.6 kilobases in length.

## Species taxonomy

Eukaryota; Metazoa; Chordata; Craniata; Vertebrata; Euteleostomi; Archelosauria; Archosauria; Dinosauria; Saurischia; Theropoda; Coelurosauria; Aves; Neognathae; Accipitriformes; Accipitridae; Accipitrinae; Accipiter; *Accipiter gentilis* (Linnaeus, 1758) (NCBI:txid8957).

## Background

The northern goshawk, *Accipiter gentilis,* is a medium-sized, forest specialist, bird of prey inhabiting large parts of the Holarctic. The considerable morphological variation particularly within *A. gentilis* has resulted in the acknowledgement of 10 subspecies ( Del Hoyo & Collar, 2015; Dickson & Remsen, 2013), with the nominate European subspecies, *A. gentilis gentilis* found across Europe, except for the Iberian Peninsula, southern Italy, and Greece, and extending eastwards to the Carpathians and part of Russia. However, mitochondrial phylogenetic analyses suggest two monophyletic groups within the species, a Neartcic clade and a Palearctic clade ( Kunz *et al*., 2019).

In the UK, loss of woodland habitat and persecution drove the species to extinction by the end of the 19 ^th^ century, before it was reintroduced during the 1960s. Despite being legally protected, *A. gentilis* are still persecuted throughout Europe ( Rutz *et al*., 2006) and their nests are frequently robbed. For instance, in Scotland, illegal killing of birds of prey in general, and northern goshawks in particular, has not declined during the last 20 years ( RSPB, 2015), leading to the hypothesis that this might have contributed to the slow recovery of the population in the UK despite repeated reintroductions ( Kenward, 2006).

## Genome sequence report

**Figure 1.**
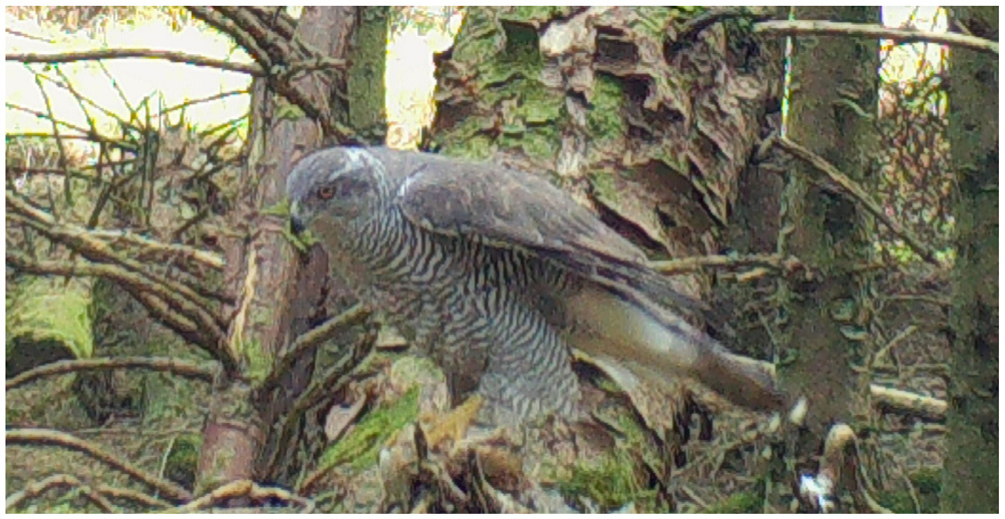
Image of the *Accipiter gentilis* specimen from the previous breeding season, captured using a camera trap.

The genome was sequenced from a single female *A. gentilis* ( Figure 1) collected from Northumberland, UK. A total of 34-fold coverage in Pacific Biosciences single-molecule HiFi long reads and 31-fold coverage in 10X Genomics read clouds were generated. Primary assembly contigs were scaffolded with chromosome conformation Hi-C data. Manual assembly curation corrected 113 missing/misjoins and removed 1 haplotypic duplication, reducing the assembly size by 0.04% and the scaffold number by 17.30%, and increasing the scaffold N50 by 9.56%.

**Figure 2.**
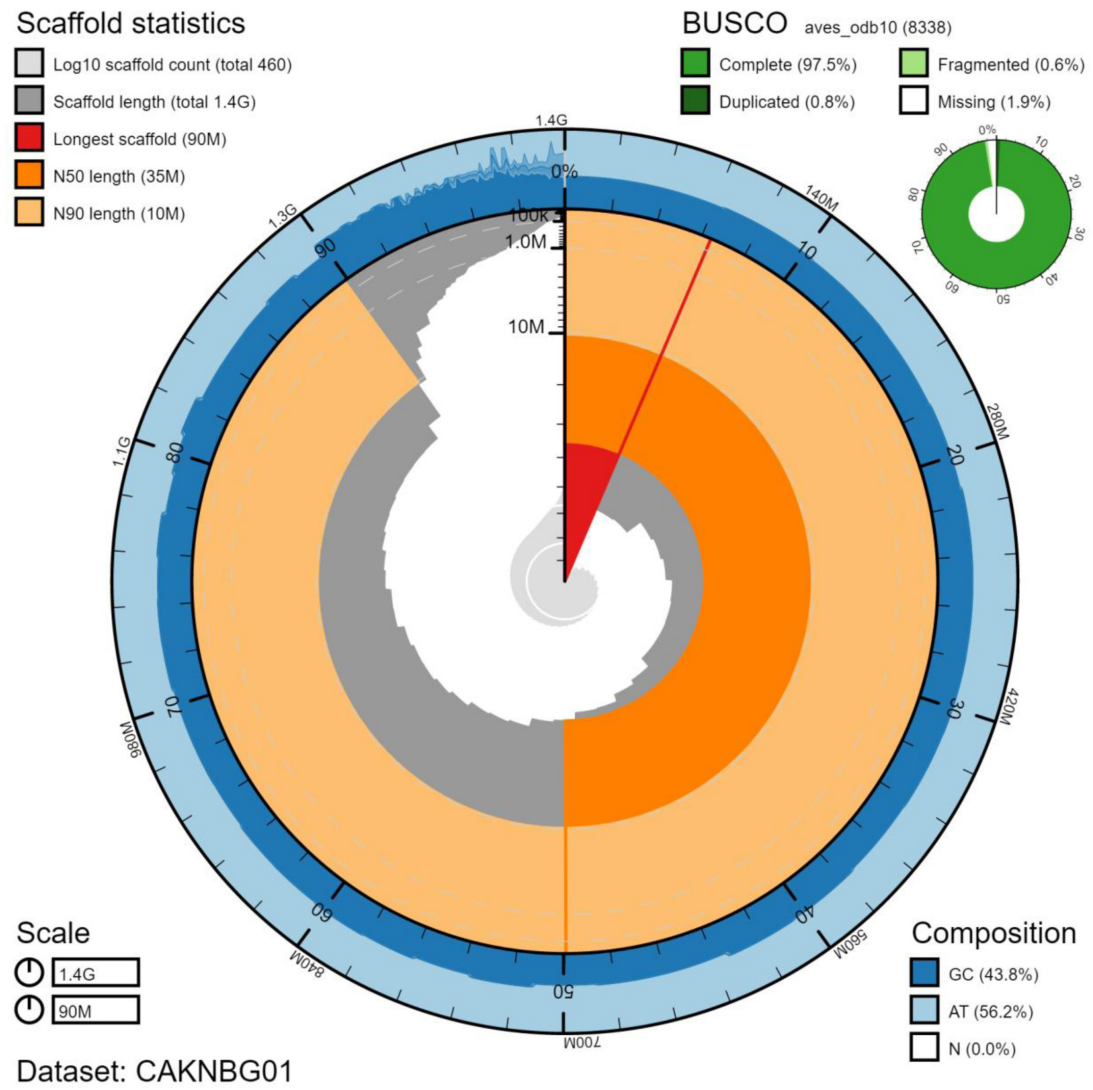
Genome assembly of *Accipiter gentilis*, bAccGen1.1: metrics. The BlobToolKit Snailplot shows N50 metrics and BUSCO gene completeness. The main plot is divided into 1,000 size-ordered bins around the circumference with each bin representing 0.1% of the 1,398,027,955 bp assembly. The distribution of scaffold lengths is shown in dark grey with the plot radius scaled to the longest scaffold present in the assembly (89,764,762 bp, shown in red). Orange and pale-orange arcs show the N50 and N90 scaffold lengths (35,025,567 and 10,404,007 bp), respectively. The pale grey spiral shows the cumulative scaffold count on a log scale with white scale lines showing successive orders of magnitude. The blue and pale-blue area around the outside of the plot shows the distribution of GC, AT and N percentages in the same bins as the inner plot. A summary of complete, fragmented, duplicated and missing BUSCO genes in the aves_odb10 set is shown in the top right. An interactive version of this figure is available at https://blobtoolkit.genomehubs.org/view/bAccGen1.1/dataset/CAKNBG01/snail.

The final assembly has a total length of 1,398 Mb in 454 sequence scaffolds with a scaffold N50 of 34.0 Mb ( Table 1). The majority, 90.67%, of the assembly sequence was assigned to 40 chromosomal-level scaffolds, representing 38 autosomes (numbered by sequence length) and the W and Z chromosomes ( Figure 2– Figure 5; Table 2). Microchromosomes 35, 36, 37, and 38 were curated based on homology to microchromosomes found in *Gallus gallus*, *Taeniopygia guttata*, and *Cuculus canorus*.

**Table 1.** Genome data for *Accipiter gentilis*, bAccGen1.1.

*Project accession data*	
Assembly identifier	bAccGen1.1
Species	*Accipiter gentilis*
Specimen	bAccGen1
NCBI taxonomy ID	8957
BioProject	PRJEB48396
BioSample ID	SAMEA8235650
Isolate information	female, heart tissue
*Raw data accessions*	
PacificBiosciences SEQUEL II	ERR7254635-ERR7254637
10X Genomics Illumina	ERR7220458-ERR7220461
Hi-C Illumina	ERR7220461
*Genome assembly*	
Assembly accession	GCA_929443795.1
*Accession of alternate haplotype*	GCA_929447715.1
Span (Mb)	1,398
Number of contigs	637
Contig N50 length (Mb)	17.7
Number of scaffolds	454
Scaffold N50 length (Mb)	35.0
Longest scaffold (Mb)	55.8
BUSCO * genome score	C:97.5%[S:96.7%,D:0.8%], F:0.6%,M:1.9%,n:8338

*BUSCO scores based on the aves_odb10 BUSCO set using v5.1.2. C= complete [S= single copy, D=duplicated], F=fragmented, M=missing, n=number of orthologues in comparison. A full set of BUSCO scores is available at https://blobtoolkit.genomehubs.org/view/bAccGen1.1/dataset/CAKNBG01/busco.

**Figure 3.**
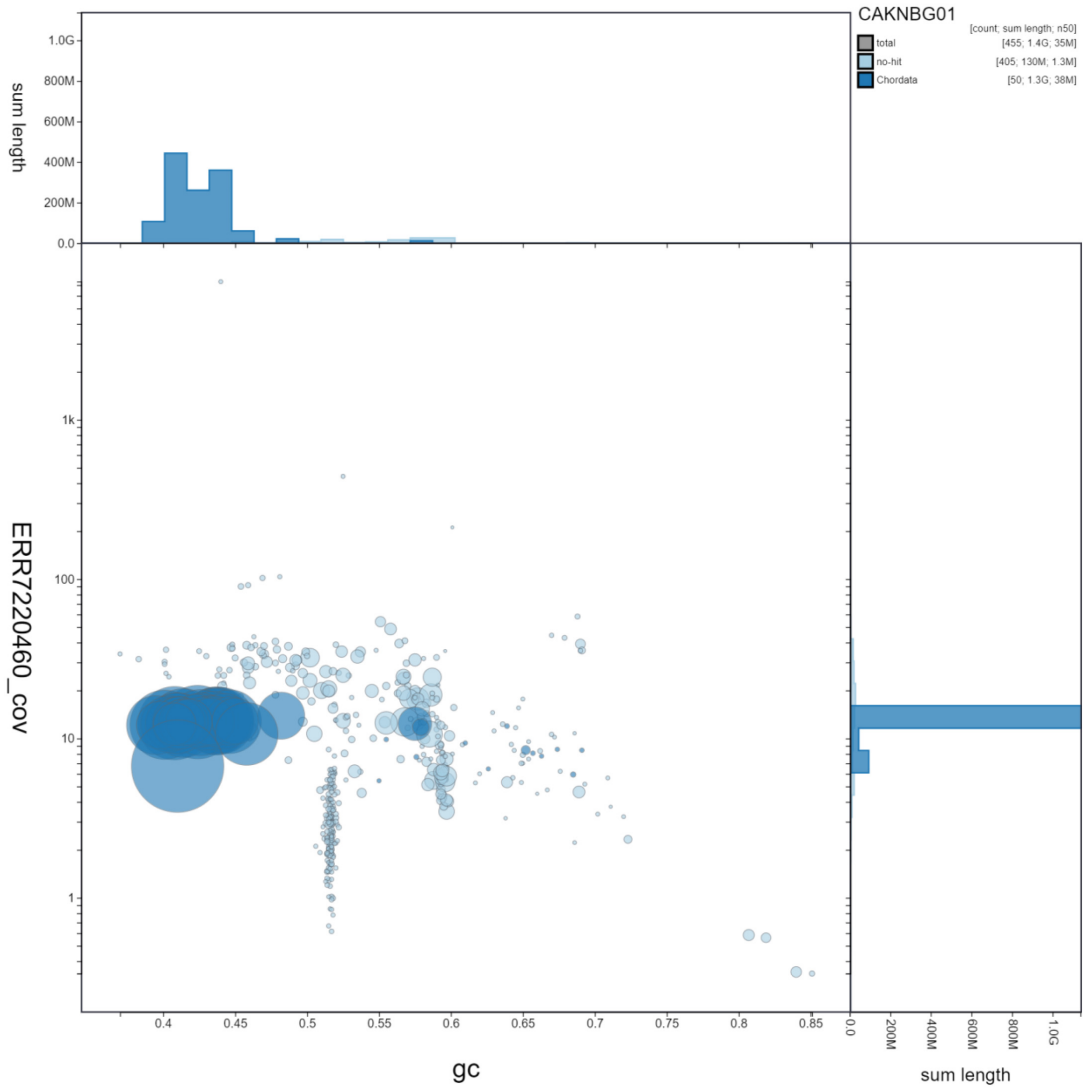
Genome assembly of *Accipiter gentilis*, bAccGen1.1: GC coverage. BlobToolKit GC-coverage plot. Scaffolds are coloured by phylum. Circles are sized in proportion to scaffold length. Histograms show the distribution of scaffold length sum along each axis. An interactive version of this figure is available at https://blobtoolkit.genomehubs.org/view/bAccGen1.1/dataset/CAKNBG01/blob.

**Figure 4.**
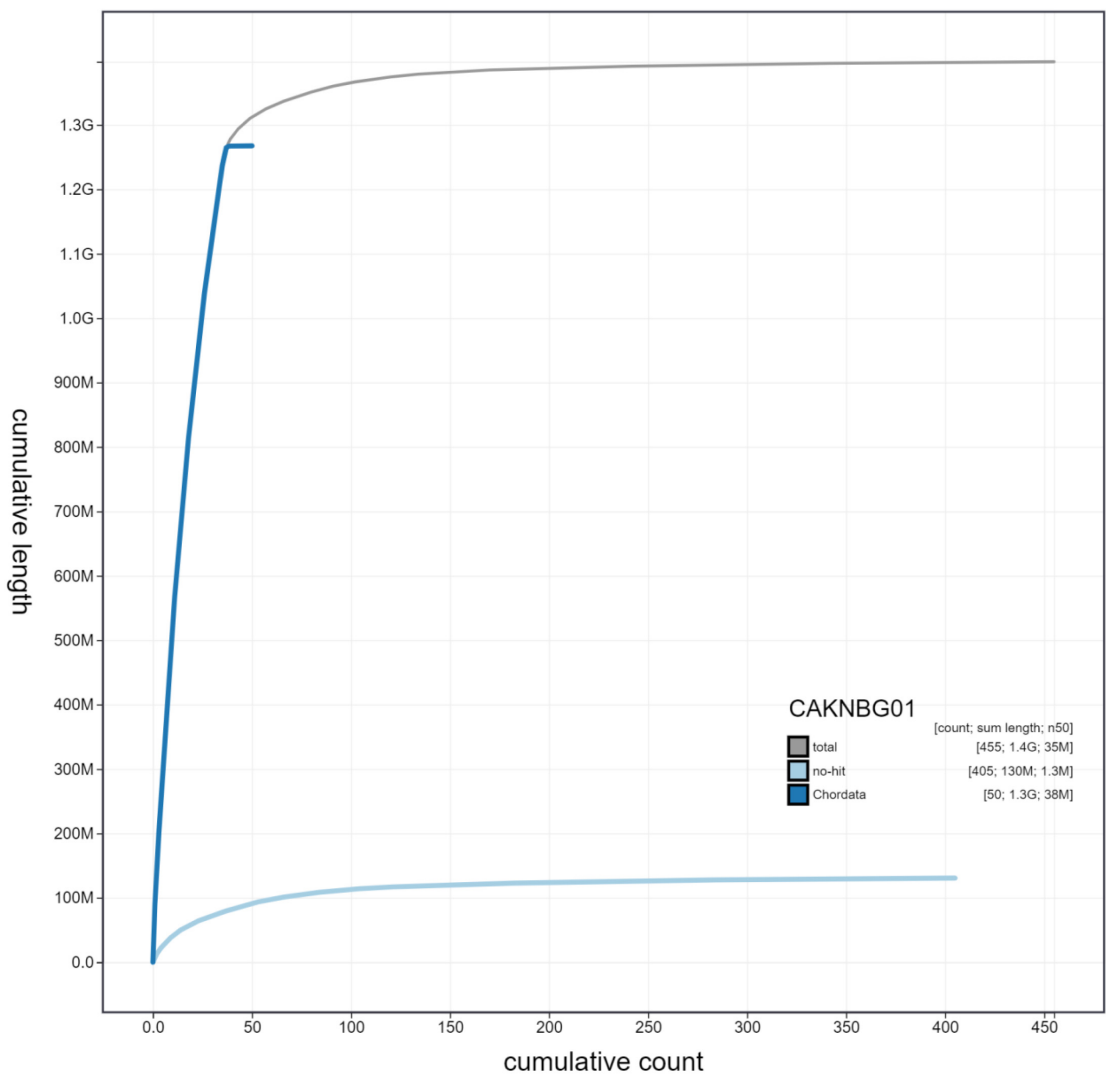
Genome assembly of *Accipiter gentilis*, bAccGen1.1: cumulative sequence. BlobToolKit cumulative sequence plot. The grey line shows cumulative length for all scaffolds. Coloured lines show cumulative lengths of scaffolds assigned to each phylum using the buscogenes taxrule. An interactive version of this figure is available at https://blobtoolkit.genomehubs.org/view/bAccGen1.1/dataset/CAKNBG01/cumulative.

**Figure 5.**
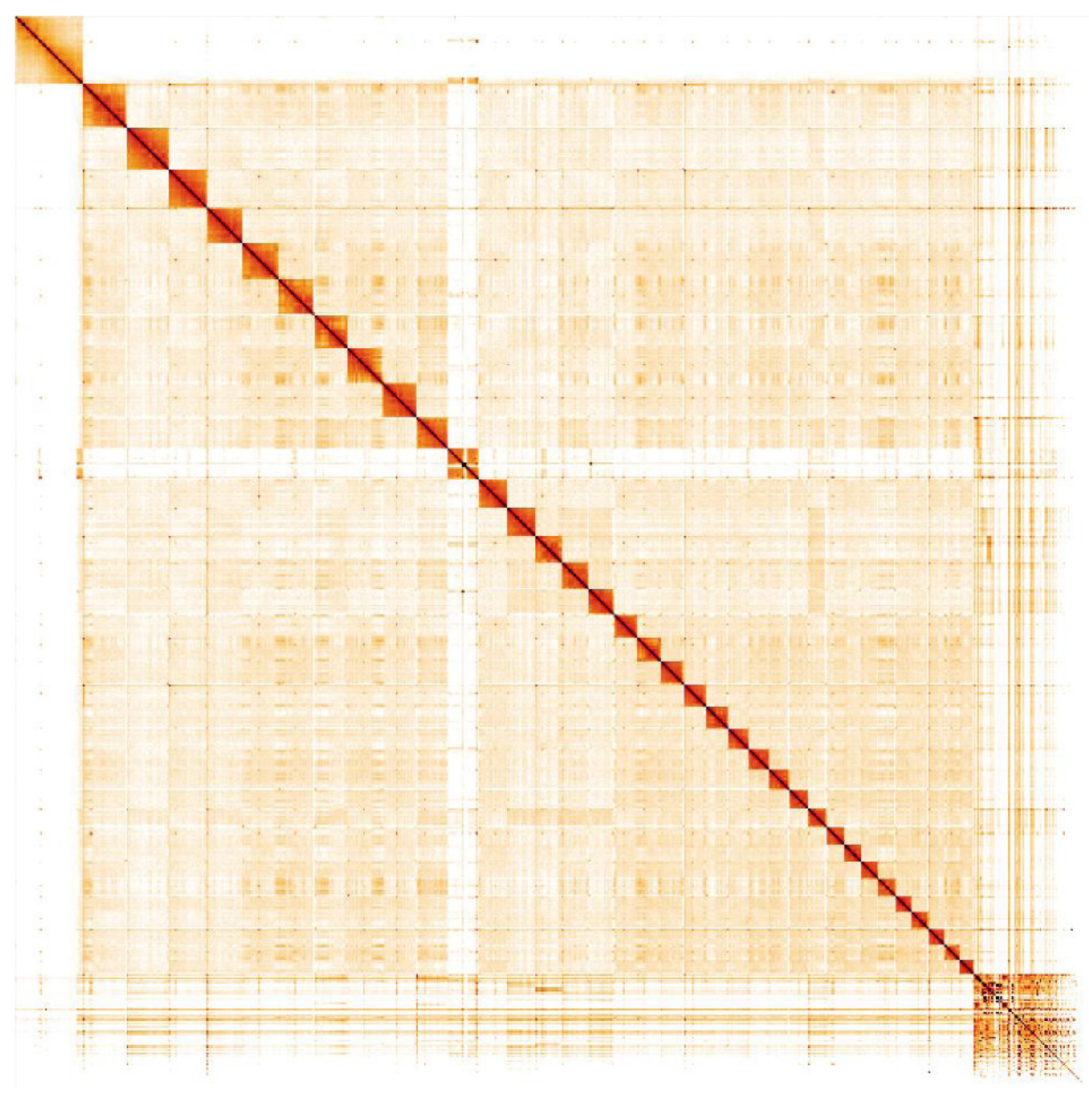
Genome assembly of *Accipiter gentilis*, bAccGen1.1: Hi-C contact map. Hi-C contact map of the bAccGen1.1 assembly, visualised in HiGlass. Chromosomes are arranged in size order from left to right and top to bottom. The interactive Hi-C map can be viewed here.

**Table 2.** Chromosomal pseudomolecules in the genome assembly of *Accipiter gentilis*, bAccGen1.1.

INSDC accession	Chromosome	Size (Mb)	GC%
OV839361.1	1	55.81	42.4
OV839362.1	2	55.41	40.8
OV839363.1	3	49.24	40.1
OV839364.1	4	48.36	41.4
OV839365.1	5	47.46	43.7
OV839366.1	6	45.22	43.7
OV839367.1	7	44.90	44.0
OV839368.1	8	44.73	43.6
OV839369.1	9	44.71	42.6
OV839370.1	10	41.28	44.5
OV839372.1	11	37.57	41.0
OV839373.1	12	37.31	40.9
OV839374.1	13	35.03	39.5
OV839375.1	14	34.43	43.4
OV839376.1	15	31.97	40.4
OV839377.1	16	31.42	42.9
OV839378.1	17	31.04	44.2
OV839379.1	18	29.66	42.7
OV839380.1	19	28.75	42.3
OV839381.1	20	28.03	40.6
OV839382.1	21	27.96	41.1
OV839383.1	22	27.20	41.1
OV839384.1	23	25.48	43.2
OV839385.1	24	25.38	43.5
OV839386.1	25	23.93	42.7
OV839387.1	26	23.47	44.6
OV839388.1	27	22.46	39.8
OV839389.1	28	21.93	43.3
OV839390.1	29	21.76	48.2
OV839391.1	30	21.43	41.5
OV839392.1	31	21.27	41.3
OV839393.1	32	21.17	41.8
OV839394.1	33	21.08	45.0
OV839395.1	34	17.59	40.8
OV839396.1	35	10.40	57.5
OV839397.1	36	2.11	57.8
OV839398.1	37	0.65	59.8
OV839399.1	38	0.43	65.1
OV839371.1	W	39.88	45.8
OV839360.1	Z	89.76	41.0
OV839400.1	MT	0.02	44.2
-	Unplaced	130.34	55.7

The assembly has a BUSCO v5.1.2 ( Manni *et al*., 2021) completeness of 97.5% (single 96.7%, duplicated 0.8%) using the aves_odb10 reference set (n=8338). While not fully phased, the assembly deposited is of one haplotype. Contigs corresponding to the second haplotype have also been deposited.

## Methods

### Sample acquisition and nucleic acid extraction

A single female *A. gentilis* specimen (bAccGen1) was found dead in coniferous woodland in Northumberland, UK by Katherine August (University of Aberdeen) and Martin Davison (Northumbria Ringing Group). The specimen was identified by Martin Davison and frozen at -20°C. Dissection of tissue samples occurred while the specimen was frozen, with the samples then stored at -80°C prior to sending to the Wellcome Sanger Institute on dry ice.

DNA was extracted at the Tree of Life laboratory, Wellcome Sanger Institute. The bAccGen1 sample was weighed and dissected on dry ice with tissue set aside for Hi-C and RNA sequencing. Heart tissue was cryogenically disrupted to a fine powder using a Covaris cryoPREP Automated Dry Pulveriser, receiving multiple impacts. Fragment size analysis of 0.01–0.5 ng of DNA was then performed using an Agilent FemtoPulse. High molecular weight (HMW) DNA was extracted using the Qiagen MagAttract HMW DNA extraction kit. Low molecular weight DNA was removed from a 200-ng aliquot of extracted DNA using 0.8X AMpure XP purification kit prior to 10X Chromium sequencing; a minimum of 50 ng DNA was submitted for 10X sequencing. HMW DNA was sheared into an average fragment size between 12–20 kb in a Megaruptor 3 system with speed setting 30. Sheared DNA was purified by solid-phase reversible immobilisation using AMPure PB beads with a 1.8X ratio of beads to sample to remove the shorter fragments and concentrate the DNA sample. The concentration of the sheared and purified DNA was assessed using a Nanodrop spectrophotometer and Qubit Fluorometer and Qubit dsDNA High Sensitivity Assay kit. Fragment size distribution was evaluated by running the sample on the FemtoPulse system.

### Sequencing

Pacific Biosciences HiFi circular consensus and 10X Genomics Chromium read cloud sequencing libraries were constructed according to the manufacturers’ instructions. Sequencing was performed by the Scientific Operations core at the Wellcome Sanger Institute on Pacific Biosciences SEQUEL II (HiFi) and Illumina NovaSeq 6000 instruments. Hi-C data were generated in the Tree of Life laboratory from remaining heart tissue of bAccGen1 using the Arima v2 kit and sequenced on a NovaSeq 6000 instrument.

### Genome assembly

Assembly was carried out with Hifiasm ( Cheng *et al*., 2021); haplotypic duplication was identified and removed with purge_dups ( Guan *et al*., 2020). One round of polishing was performed by aligning 10X Genomics read data to the assembly with longranger align, calling variants with freebayes ( Garrison & Marth, 2012). The assembly was then scaffolded with Hi-C data ( Rao *et al*., 2014) using yahs. The assembly was checked for contamination as described previously ( Howe *et al*., 2021). Manual curation was performed using HiGlass ( Kerpedjiev *et al*., 2018) and Pretext. The mitochondrial genome was assembled using MitoHiFi ( Uliano-Silva *et al*., 2021), which performs annotation using MitoFinder ( Allio *et al*., 2020). The genome was analysed and BUSCO scores generated within the BlobToolKit environment ( Challis *et al*., 2020). Table 3 contains a list of all software tool versions used, where appropriate.

**Table 3.** Software tools used.

Software tool	Version	Source
Hifiasm	0.15.3	Cheng *et al*., 2021
purge_dups	1.2.3	Guan *et al*., 2020
yahs	1.0	https://github.com/c-zhou/yahs
longranger align	2.2.2	https://support.10xgenomics.com/ genome-exome/software/pipelines/ latest/advanced/other-pipelines
freebayes	1.3.1-17- gaa2ace8	Garrison & Marth, 2012
MitoHiFi	2.0	Uliano-Silva *et al*., 2021
HiGlass	1.11.6	Kerpedjiev *et al*., 2018
PretextView	0.2.x	https://github.com/wtsi-hpag/ PretextView
BlobToolKit	3.0.5	Challis *et al*., 2020

### Ethics/compliance issues

The materials that have contributed to this genome note have been supplied by a Tree of Life collaborator. The Wellcome Sanger Institute employs a process whereby due diligence is carried out proportionate to the nature of the materials themselves, and the circumstances under which they have been/are to be collected and provided for use. The purpose of this is to address and mitigate any potential legal and/or ethical implications of receipt and use of the materials as part of the research project, and to ensure that in doing so we align with best practice wherever possible.

The overarching areas of consideration are:

- Ethical review of provenance and sourcing of the material
- Legality of collection, transfer and use (national and international)

Each transfer of samples is undertaken according to a Research Collaboration Agreement or Material Transfer Agreement entered into by the Tree of Life collaborator, Genome Research Limited (operating as the Wellcome Sanger Institute) and in some circumstances other Tree of Life collaborators.

## Data availability

European Nucleotide Archive: Accipiter gentilis (northern goshawk). Accession number PRJEB48396; https://identifiers.org/ena.embl/PRJEB48396.

The genome sequence is released openly for reuse. The *A. gentilis* genome sequencing initiative is part of the Darwin Tree of Life (DToL) project. All raw sequence data and the assembly have been deposited in INSDC databases. The genome will be annotated and presented through the Ensembl pipeline at the European Bioinformatics Institute. Raw data and assembly accession identifiers are reported in Table 1.

## Author information

Members of the Wellcome Sanger Institute Tree of Life programme are listed here: https://doi.org/10.5281/zenodo.6125027.

Members of Wellcome Sanger Institute Scientific Operations: DNA Pipelines collective are listed here: https://doi.org/10.5281/zenodo.5746904.

Members of the Tree of Life Core Informatics collective are listed here: https://doi.org/10.5281/zenodo.6125046.

Members of the Darwin Tree of Life Consortium are listed here: https://doi.org/10.5281/zenodo.5638618.
