# Supplementary material for: The genome sequence of the northern goshawk, Accipiter gentilis (Linnaeus, 1758)
Source: Wellcome Open Res. 2022 Apr 4;7:122. [Version 1] doi: 10.12688/wellcomeopenres.17821.1 (PMC9020535; doi:10.12688/wellcomeopenres.17821.1)
Supplement: Peer review history

# Peer review: Reviewer response for version 1

**DOI:** 10.21956/wellcomeopenres.19730.r49691

**Published:** 2022-04-19

**Authors:** Darren Irwin

**Affiliations:** 1 Department of Zoology, Biodiversity Research Centre, University of British Columbia, Vancouver, Canada

This is a mostly clear communication of what appears to be an excellent genome assembly for the Northern Goshawk. A combination of state-of-the-art methods were used, leading me to have high confidence in the quality of the assembly. This will be very useful for the community of researchers studying goshawks and other raptors, as well as for those interested in methods of producing excellent chromosome-scale reference genomes.

 I have no major concerns and approve of this version as being acceptable for full publication.

 I do however have a few suggestions that the authors might want to consider:

 In the second paragraph under “Background”, the paper mentions that goshawks were extirpated from the UK and then reintroduced. It would be useful for readers to be told: where were the reintroduced individuals from? This is relevant to readers who want to understand the genetic origin(s) of the new UK population (since there is geographic variation in goshawks elsewhere). Also, a technical quibble: the term “extinction” (line two of the second paragraph) is usually only used for cases where the species is gone from the entire world—in this case “extirpation” or “locally extinct” would be more accurate.

 I would have been interested in learning more about the manual assembly curation, and the causes of misjoins in the initial assembly (e.g., what software produced them, and why?)

 As a relatively new reader of papers such as this that present genome assemblies, I found Fig. 2 and Fig. 3 challenging to understand. After going to the external link provided for Fig. 2 and playing with it there, I now understand most of Fig. 2. However, the caption refers to a “log scale” for the “cumulative scaffold count” of the “grey spiral”—if this is the same as the scale down the top middle, it looks like there is differing spatial distance between 100K to 1.0M compared to 1.0M to 10M, whereas on a log scale I would think those should be the same.

 After looking at the external link for Fig. 3, I am still not sure of what that figure shows. What does the caption mean when it refers to “coloured by phylum”? Why do the histograms not seem to match the scatter plot well?

 My overall suggestion for those two figures would be to revise the captions so they are more understandable to those not familiar with such figures, and to more clearly explain what the reader should infer from these figures.

 Those questions aside, I think this paper is an excellent contribution and will be widely appreciated by those in a position to make use of this genome assembly.

Are sufficient details of methods and materials provided to allow replication by others?

Yes

Is the rationale for creating the dataset(s) clearly described?

Yes

Are the datasets clearly presented in a useable and accessible format?

Yes

Are the protocols appropriate and is the work technically sound?

Yes

Reviewer Expertise:

evolution; hybridization; genomic differentiation; speciation; birds

I confirm that I have read this submission and believe that I have an appropriate level of expertise to confirm that it is of an acceptable scientific standard.

# Peer review: Reviewer response for version 1

**DOI:** 10.21956/wellcomeopenres.19730.r49693

**Published:** 2022-04-05

**Authors:** Jong Bhak

**Affiliations:** 1 Ulsan National Institute of Science and Technology (UNIST), Ulsan, South Korea

The authors present a genome assembly of Accipiter gentilis (goshawk). 

 The quality of the assembly is very high (PacBio) and this is an important genomic resource for avian genomics.

 This reviewer finds it all very properly sequenced and analyzed with the latest tools. 

 I guess the annotation of the raptor will be done automatically by Ensembl pipeline. Therefore, biological analyses are out of the scope of this paper.

 I recommend it to be accepted without delay.

Are sufficient details of methods and materials provided to allow replication by others?

Yes

Is the rationale for creating the dataset(s) clearly described?

Yes

Are the datasets clearly presented in a useable and accessible format?

Yes

Are the protocols appropriate and is the work technically sound?

Yes

Reviewer Expertise:

Genome assembly. Genome annotation. Bioinformatics. RNA-seq analyses. Long read sequencing.

I confirm that I have read this submission and believe that I have an appropriate level of expertise to confirm that it is of an acceptable scientific standard.
